# The HIV-1 Transactivator Factor (Tat) Induces Enterocyte Apoptosis through a Redox-Mediated Mechanism

**DOI:** 10.1371/journal.pone.0029436

**Published:** 2011-12-27

**Authors:** Vittoria Buccigrossi, Gabriella Laudiero, Emanuele Nicastro, Erasmo Miele, Franca Esposito, Alfredo Guarino

**Affiliations:** 1 Department of Paediatrics, University of Naples “Federico II,” Naples, Italy; 2 Department of Biochemistry and Medical Biotechnology, University of Naples “Federico II,” Naples, Italy; German Primate Center, Germany

## Abstract

The intestinal mucosa is an important target of human immunodeficiency virus (HIV) infection. HIV virus induces CD4+ T cell loss and epithelial damage which results in increased intestinal permeability. The mechanisms involved in nutrient malabsorption and alterations of intestinal mucosal architecture are unknown. We previously demonstrated that HIV-1 transactivator factor (Tat) induces an enterotoxic effect on intestinal epithelial cells that could be responsible for HIV-associated diarrhea. Since oxidative stress is implicated in the pathogenesis and morbidity of HIV infection, we evaluated whether Tat induces apoptosis of human enterocytes through oxidative stress, and whether the antioxidant N-acetylcysteine (NAC) could prevent it. Caco-2 and HT29 cells or human intestinal mucosa specimens were exposed to Tat alone or combined with NAC. In an *in-vitro* cell model, Tat increased the generation of reactive oxygen species and decreased antioxidant defenses as judged by a reduction in catalase activity and a reduced (GSH)/oxidized (GSSG) glutathione ratio. Tat also induced cytochrome c release from mitochondria to cytosol, and caspase-3 activation. Rectal dialysis samples from HIV-infected patients were positive for the oxidative stress marker 8-hydroxy-2′-deoxyguanosine. GSH/GSSG imbalance and apoptosis occurred in jejunal specimens from HIV-positive patients at baseline and from HIV-negative specimens exposed to Tat. Experiments with neutralizing anti-Tat antibodies showed that these effects were direct and specific. Pre-treatment with NAC prevented Tat-induced apoptosis and restored the glutathione balance in both the *in-vitro* and the *ex-vivo* model. These findings indicate that oxidative stress is one of the mechanism involved in HIV-intestinal disease.

## Introduction

The intestinal mucosa is a functional barrier against pathogens being both a physical obstacle with columnar cells linked together by tight junctions, and the site of mucosal immunological cells. HIV infection is mainly initiated on the intestinal mucosal surface through heterosexual or homosexual transmission [1], [2] and HIV acutely induces infiltration of the gut mucosa thereby resulting in the release of activated effector memory CD4+ and CD8+ T cells, damage to the intestinal barrier and increased epithelial apoptosis [3]. Clinical data support a relationship between chronic HIV infection and intestinal dysfunction including increased permeability, altered nutrient absorption, diarrhea and reduction of the absorptive surface [4]–[10]. Acquired immunodeficiency syndrome (AIDS) enteropathy is an idiopathic, pathogen-negative diarrhea and is associated with an increase in inflammation [11], mucosal immune activation, villous atrophy and crypt hyperplasia that may be observed in all stages of HIV disease even in the absence of HIV virus [12]. The detection of viral proteins and/or nucleic acids in enterocytes and in goblet cells indicated that HIV virus plays a direct pathogenic role at intestinal level [13], [14]. Kotler et al. detected HIV DNA, RNA and protein antigens in lamina propria mononuclear cells and epithelial cells of gastrointestinal tract from HIV patients [14].

However, several effects induced by HIV are not mediated by lytic propagation of viral particles, but rather by viral factors that are released by infected cells [15]. We previously demonstrated that the viral protein Tat induces ion secretion in Caco-2 cells and in human colonic mucosa, and inhibits intestinal cell proliferation. Tat-induced ion secretion is associated with an increase in intracellular Ca^2+^ as a result of extracellular Ca^2+^ entrance and mobilization of intracellular stores [16]. A similar effect is induced by Tat in neurons [17]. In addition, Tat causes an imbalance in reactive oxygen species (ROS) generation in neurons, which is neutralized by antioxidants, thereby implicating perturbation of the intracellular redox status in the pathogenesis of HIV-induced cell damage [18].

Oxidative stress is implicated in the pathogenesis and morbidity of HIV infection [19], [20]. An increase of ROS and an alteration of antioxidant defenses have been reported in HIV-infected patients [21] associated with decreased levels of antioxidants [22]. The mechanisms involved in HIV-induced oxidative stress are unknown, but HIV-1 proteins gp120 and Tat have been implicated in this process [23] because both induce oxidative stress and cause apoptosis in brain endothelial cells [23].

Antioxidant defenses are also impaired in HIV-infected patients and, in particular, glutathione metabolism is altered [24]. Reduced glutathione (GSH) is the main intracellular thiol molecule responsible for ROS scavenging and for the maintenance of oxidative balance. It is also involved in the protection of DNA and nuclear proteins from oxidative damage. Intracellular GSH depletion triggers ROS production thereby inducing an arrest in the intestinal cell cycle [25]. GSH levels are depleted in plasma, in epithelial lining fluid of the lower respiratory tract, in peripheral blood mononuclear cells and in monocytes in HIV-infected patients [26]. Antioxidant deficiency leads to severe degeneration of intestinal epithelial cells, and even a mild intracellular redox imbalance inhibits enterocyte proliferation [27].

Interestingly, GSH levels progressively decrease as the HIV-1 viral load increases [28]. A fall in GSH during HIV infection may result from reduced GSH synthesis or increased catabolism. Tat blocks transcription of manganese superoxide dismutase, an enzyme that prevents oxidative stress, and decreases the activity of glucose-6-phosphate dehydrogenase, a key enzyme in pathways that maintain GSH in its reduced state [29]. Moreover, Tat induced oxidative stress in an immortalized endothelial cell line from rat brain capillaries [30].

In this scenario, we hypothesized that the enteropathogenic effects induced by Tat are associated with an imbalance of the redox state in the intestine.

## Results

### HIV-1 Tat induces intestinal epithelial oxidative stress thereby increasing reactive oxygen species and impairing antioxidant defenses

**Figure 1:**
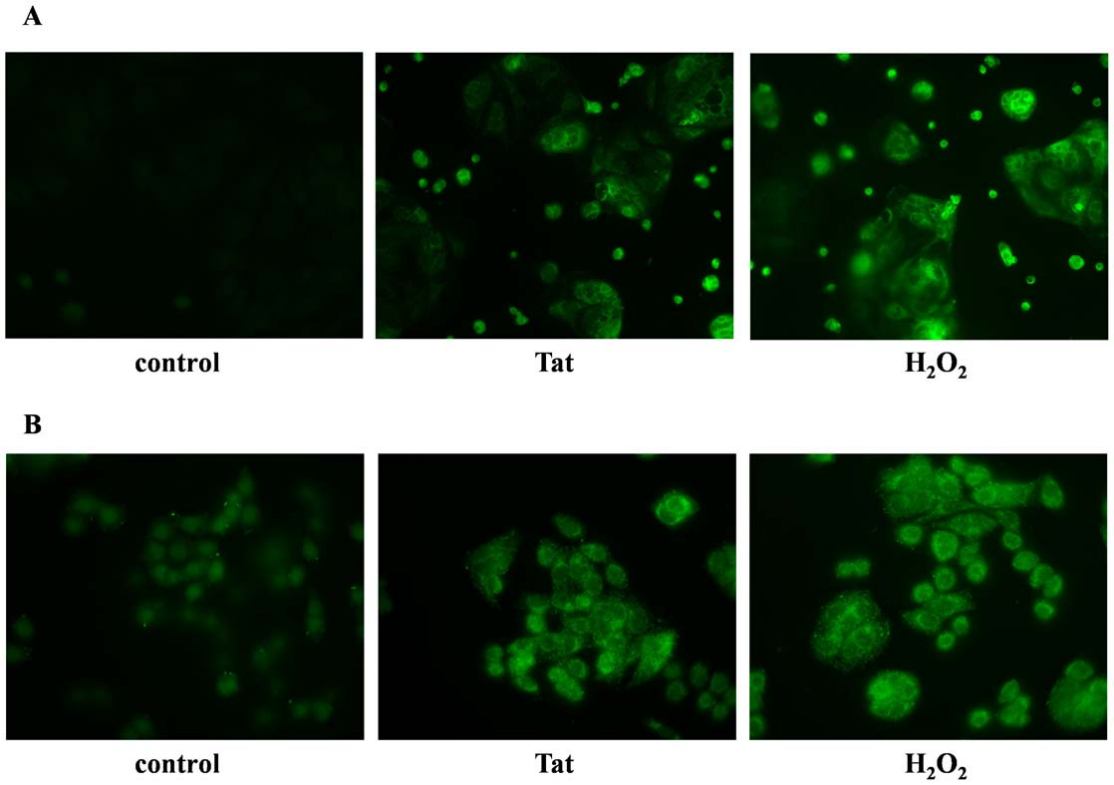
Influence of HIV-Tat protein on ROS generation in Caco-2 cells (A) and HT29 cells (B). Immunofluorescent staining of ROS by DCFH-DA after Tat exposure were compared to H_2_O_2_- and untreated cells (control). Representative staining is shown at 1 hour post-exposure. Magnification: 200×. Data are representative of 3 separate experiments with 3–4 replicates for each experimental condition.

**Figure 2:**
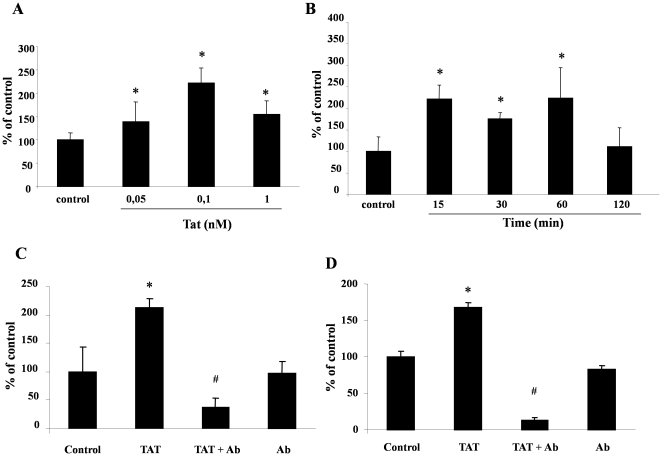
Tat-induced ROS generation is dose- and time-dependent. Caco-2 cells were exposed to different concentrations of Tat for 1 hour (A) and to 0.1 nM for 10, 30, 60 and 120 min (B), and ROS intracellular levels were evaluated by the DCFH-DA fluorometric method. To evaluate the specificity of the effect, Caco-2 cells (C) and HT-29 (D) were incubated with Tat with or without the anti-Tat polyclonal antibody. Data are representative of 3 separate experiments. *p<0,05 vs control; #p<0,05 vs Tat.

To evaluate whether an altered redox state could be responsible for the effects induced by Tat, we measured the intracellular levels of ROS and of two main intracellular antioxidant defense systems, catalase and glutathione, in intestinal epithelial cells. Fluorescence microscopy showed that ROS levels were increased in Caco-2 cells exposed to 0.5 nM of Tat for 1 hour as judged by the fluorescence green signal produced by the interaction between dichlorodihydrofluorescein diacetate (DCFH-DA) and ROS (Fig. 1A). ROS production was also increased in HT-29 cells stimulated with HIV-Tat under the same conditions (Fig. 1B). As a positive control, cells were treated with H_2_O_2_, and cells treated with the same volume of media without Tat protein served as negative control. Increasing Tat concentrations (0.05–1 nM) were added to Caco-2 cells. DCFH-DA was used for ROS quantification and measured 15 min after Tat stimulation. Exposure to Tat protein resulted in a dose-dependent increase of ROS (Fig. 2A). Since ROS generation is usually rapid after a toxic stimulus, we performed time-course experiments in Caco-2 cells exposed to Tat for 15, 30, 60 and 120 min (Fig. 2B). A ROS increase was evident as early as 15 min after exposure to Tat; levels returned to control values after two hours. This suggests that the antioxidant defenses may be activated to counteract oxidative stress.

We carried out neutralization experiments to ascertain whether ROS generation is a specific effect induced by Tat. As shown in Fig. 2C and D, anti-Tat polyclonal antibodies inhibited the increase of ROS intracellular levels in Caco-2 and HT-29 cells. To see whether Tat enters into the cells, we stimulated Caco-2 cells from the same culture with Tat at 1, 24, 48 and 72 hrs and, in parallel, we evaluated ROS concentrations and Tat intracellular levels (Fig. S1). In these conditions, Tat was detected inside the cells 1 hour after stimulation and until at least 72 hours post-exposure (Fig. S1C).

**Figure 3:**
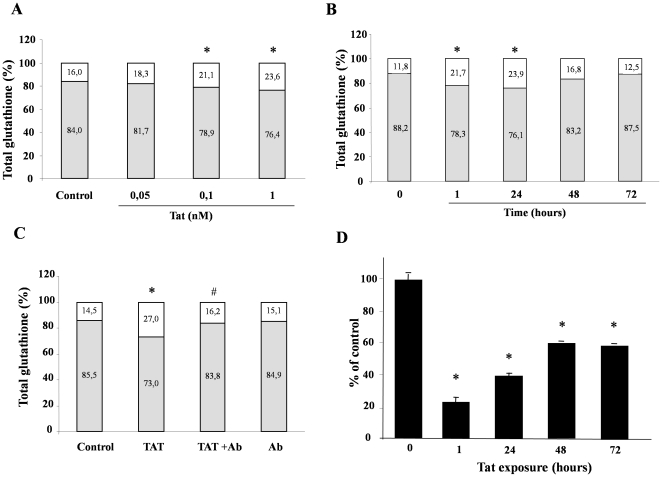
Tat induced alteration of intracellular antioxidant defenses. Caco-2 cells were exposed to different concentrations of Tat for 1 hour (A) and to 0.1 nM for 10, 30, 60 and 120 min (B), and the percent of GSH (grey) and GSSG (white) was evaluated as described under “Methods” in the Methods section. To evaluate the specificity of the effect, Caco-2 cells (C) were incubated with Tat with or without the anti-Tat polyclonal antibody. *p<0,05 vs control; #p<0,05 vs Tat. (D) Catalase activity was evaluated in Caco-2 cells after different times of Tat exposure. Data are representative of 3 separate experiments. *p<0,05 vs time 0.

We next investigated whether Tat-induced ROS generation was associated with a decrease of antioxidant defenses by measuring the levels of glutathione, one of the major intracellular ROS scavengers. Glutathione is an important cellular antioxidant, and it plays a major role in protecting cells against oxidative stress. In fact, the intracellular balance between the reduced (GSH) and oxidated (GSSG) glutathione forms in healthy conditions was reported to show a predominantly reducing state being GSH about 80–90% and GSSG 10–20% [31]. We found that the GSH/GSSG ratio was lower in Caco-2 cells exposed to Tat than in controls and that the effect was dose- and time-dependent (Fig. 3A and B). The reduction of GSH was significant between 0.1 and 1 nM of Tat (Fig. 3A) and was already evident at 1 hour post-exposure. GSH remained low at 24 hours and returned to basal level at 48–72 hours (Fig. 3B). Also in this case, anti-Tat polyclonal antibodies completely inhibited the Tat-induced GSH/GSSG imbalance in Caco-2 (Fig. 3C) and in HT-29 cells (Fig. S2). The decreased activity of the enzyme catalase, another component of the antioxidant defense, after 1 hour of exposure to Tat (Fig. 3D) indicates that antioxidant defense is a target of Tat.

### Intestinal oxidative stress is increased in HIV-infected children

**Figure 4:**
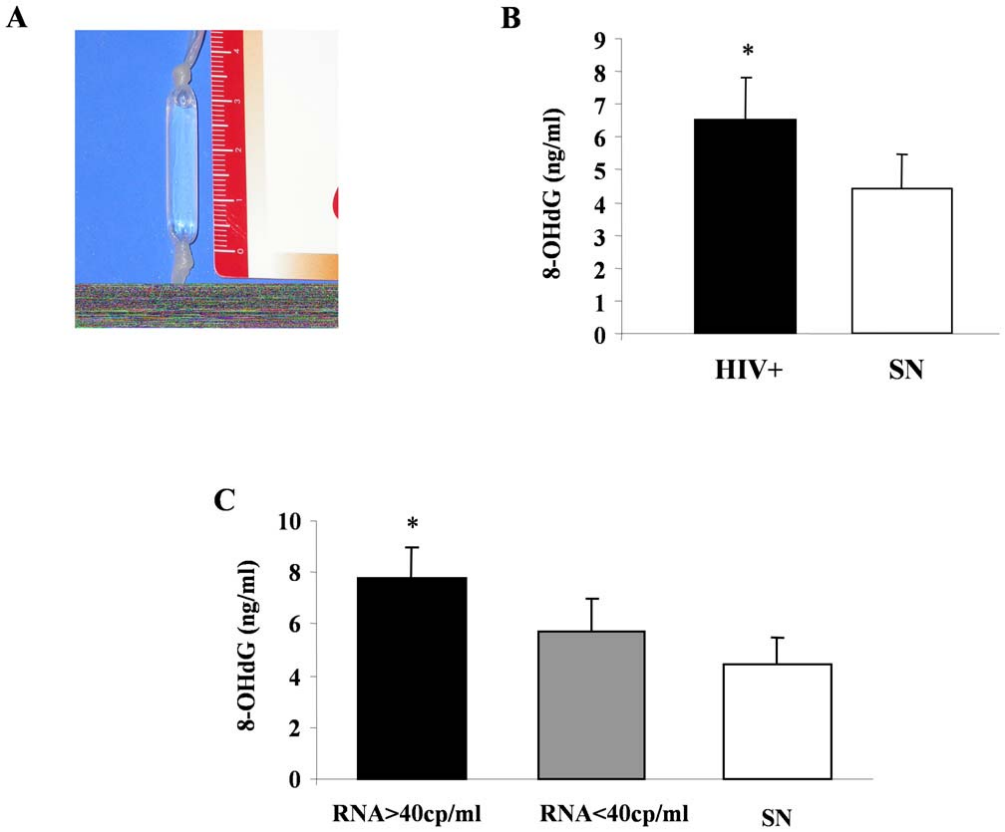
8-hydroxy-2′-deoxyguanosine (8-OHdG) concentration in rectal dialysis in HIV-negative and positive children. The determinations of rectal 8-OHdG production were performed using a dialysis bag (A) in serum-negative (n = 6, SN) and positive (n = 20, HIV+) children (B). *p<0.05 vs SN. (C) HIV+ children were divided in two groups and compared with controls (n = 6, white): HIV RNA >40cp/ml (n = 9, black), HIV RNA <40cp/ml (n = 11, grey). *p<0.05 vs RNA <40cp/ml and SN groups.

In nuclear and mitochondrial DNA, 8-hydroxy-2′-deoxyguanosine (8-OHdG) is one of the predominant marker of free radical-induced oxidative damage and is therefore widely used as biomarker for oxidative stress in clinical samples [32]–[34]. To investigate whether HIV-infected patients are affected by intestinal oxidative stress, we measured 8-OHdG concentration in a small amount of rectal dialysis solution from 20 HIV-positive children treated in our tertiary care center for patients with AIDS (Fig. 4A). The characteristics of patients are shown in Table 1. The concentration of 8-OHdG was higher in HIV-infected children than in serum-negative children (Fig. 4B). To investigate whether intestinal oxidative stress was related to virus replication, we divided the HIV-positive patients in two groups related to plasma viral load. We identified 9 children with a viral load higher than 40 copies/ml and 11 children with undetectable HIV RNA (<40 copies/ml). The mean 8-OHdG concentration was higher in children with HIV RNA >40 copies/ml than in children with HIV RNA <40 copies/ml and in serum-negative children (Fig. 4C), which suggests that in children with chronic HIV infection, oxidative damage measured in rectal mucosa may be linked with viral replication. However, 8-OHdG concentrations in urine and serum did not differ between patients and HIV-negative control children (Fig. S3), suggesting that the oxidative stress is localized in rectal mucosa. Finally, a significant correlation was detected between 8-OHdG and viral load (r = 0.4653; p = 0.0009).

**Table 1:** Characteristics of the HIV-positive patients.

**Age (months)**	
Mean ± standard deviation	134.8±53.3
Range	26–228
**Sex**	
Male	6
Female	14
**CD4+ (cells/ml)**	
Mean ± standard deviation	963.85±496.43
Range	176–2027
**HIV RNA**	
Patients with HIV RNA <40cp/ml	11
Patients with HIV RNA >40cp/ml	9
Mean viral load (cp/ml)± standard deviation	3934.89±6776.77
Range	48–14500

### Activation of caspase-3 and cytochrome c release in the cytosol indicates that Tat induces apoptosis through involvement of the intrinsic pathway

**Figure 5:**
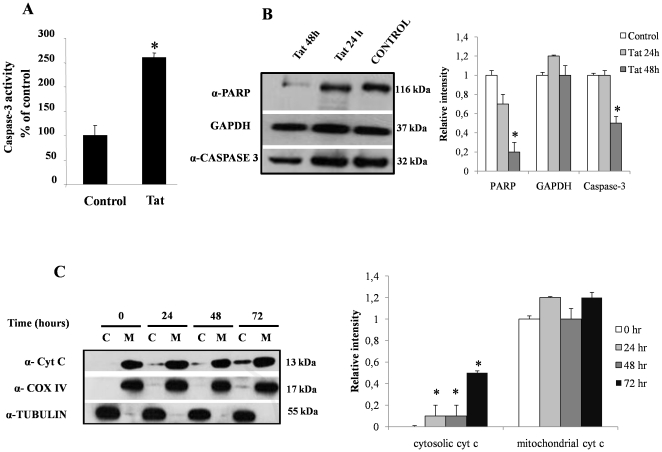
Influence of HIV-Tat protein on apoptosis in Caco-2 cells. Caspase-3 activity (A) and full-length protein (B lower panel) were evaluated in Tat-treated Caco-2 cells. To verify that Tat induced apoptosis, cleaved PARP was evaluated in the same western blot used to evaluate the activation of caspase-3 (B upper panel). Normalization of western blot was performed with GAPDH in all experiments (B middle panel). *p<0.05 compared with untreated control cells. Protein extracts from cytosol (C lanes) and mitochondria (M lanes) assayed for cytochrome c by western blot analysis (C); tubulin was used as cytosolic marker, and COX IV as mitochondrial marker. Densitometric acquisitions are shown from three separate experiments. *p<0,05 vs time 0.

Several viruses induce apoptosis as a strategy to spread the infection or to induce the breakdown of infected cells, thereby favoring viral dissemination. To investigate whether Tat induces intestinal apoptosis, we studied caspase-3 signaling, which is activated by two fundamentally distinct signaling cascades, namely the extrinsic and intrinsic (or mitochondrial) pathways [35]. We found that caspase-3 activity was higher in Caco-2 cells exposed to Tat for 72 hours than in control cells (Fig. 5A). Caspase-3 is a critical effector of apoptosis and is responsible for the proteolytic cleavage of many key proteins including the nuclear enzyme poly (ADP ribose) polymerase (α-PARP). In a western blot time-course experiment, we found that the levels of the full-length forms of caspase-3 protein and α-PARP were reduced after Tat exposure compared with control cells thereby indicating activation of the apoptotic molecular mechanisms (Fig. 5B).

Proapoptotic stimuli, including ROS and calcium overload, are able to activate the intrinsic pathway of apoptosis by inducing mitochondrial membrane permeabilization and the release of cytochrome c in the cytosol. Because we previously found that Tat induced an increase of cytosolic calcium from intracellular stores [16], we used western blot analysis to test the effects of Tat on the release of cytochrome c. Twenty-four hours after Tat exposure, cytochrome c levels in the cytosol of cells were very high and they continued to increase up to 72 hours (Fig. 5C upper panel). Cytochrome c was not detected in the cytosol of control cells. The same filter was reprobed with a monoclonal antibody against cytochrome oxidase subunit IV (COX IV), which is a mitochondrial marker, and then with anti-tubulin antibody, which is a cytosolic marker. The absence of COX IV in the cytosolic samples confirmed the absence of mitochondrial contamination in the cytosolic fraction (Fig. 5C middle panel), and the absence of tubulin in mitochondrial samples indicates that the mitochondrial fractions were free from cytosolic content (Fig. 5C lower panel).

**Figure 6:**
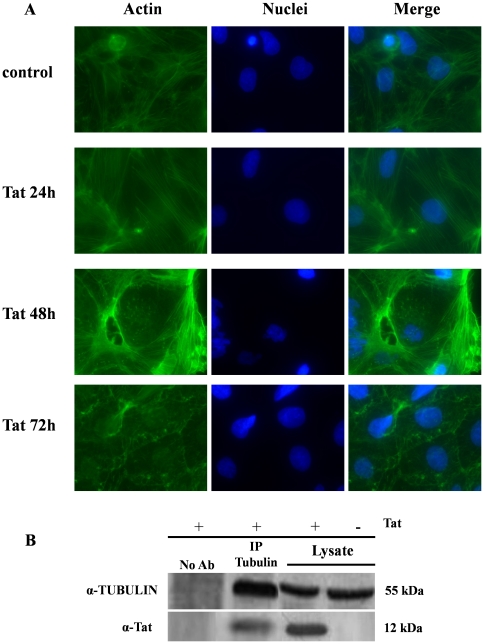
Tat-induced disruption of the actin cytoskeleton. (A) Direct immunofluorescence of actin staining by FITC-conjugated phalloidin (green). The nuclei were stained with Hoerst (blue). Caco-2 cells were exposed to Tat 0.5 nM for 72 hours. Magnification: 1000×. Data are representative of 3 separate experiments with 3–4 replicates for each experimental condition. (B) Cell lysates of Caco-2 cells were stimulated with Tat 0,5 nM for 24 hours and subjected to immunoprecipitation using anti-tubulin antibody or control IgG. Tat protein was detected from immunoprecipitates by Western blotting with anti-Tat antibody.

Because oxidative stress disrupts the cytoskeleton, we investigated the intracellular actin architecture. The architecture of actin cytoskeleton was normal in Caco-2 cells, but it became instable and fragmented after Tat exposure. Disruption of the actin cytoskeleton was dependent on the time of Tat exposure (Fig. 6A). To investigate the effects of Tat on microtubules in Caco-2 cells, we performed experiments of co-immunoprecipitation of Tat and tubulin. The protein extracts from Caco-2 cells exposed to Tat were subjected to co-immunoprecipitation with an antibody against tubulin or with control IgG, and subsequently analyzed through Western blotting with the anti-Tat polyclonal antibody. Tat coprecipitated with tubulin indicating that it binds to the microtubule cytoskeleton (Fig. 6B).

### Polar effects by Tat on the apical and basolateral side of the Caco-2 cell monolayers

**Figure 7:**
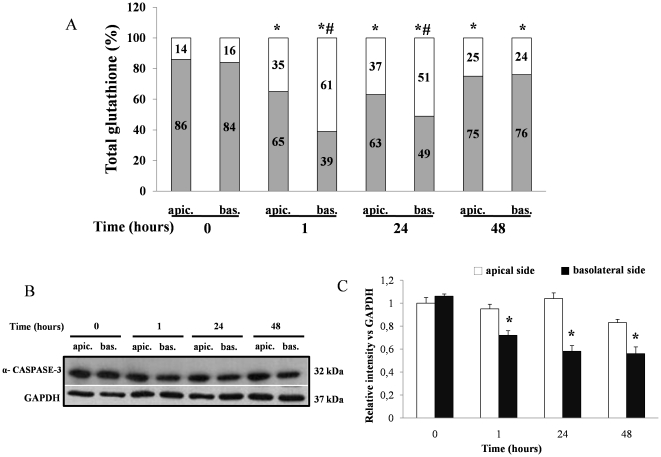
Polar effects by Tat on the apical and basolateral side of the Caco-2 cell monolayers. Tat was added to apical or basolateral side of Caco-2 cell monolayers and GSH (grey)/GSSG (white) ratio (A) and caspase-3 activation (B) were evaluated after 1, 24 and 48 hours. *p<0,05 vs control; #p<0,05 vs apical Tat stimulation at the same time. Normalization of western blot was performed with GAPDH in all experiments. Densitometric acquisitions are shown from three representative separate experiments.

To test the hypothesis that Tat induces different effects depending on its addition to the apical or basolateral side of the epithelium, Caco-2 cell monolayers were exposed to Tat 0.5 nM for 1, 24 and 48 hours at apical or basolateral side (Fig. 7). In these conditions Tat reduced the GSH/GSSG ratio at 1–24 hours, with a more potent effect at the basolateral than the apical side (Fig. 7A). In addition, the activation of caspase-3 was observed following basolateral but not apical stimulation (Fig. 7B). The polar effect by Tat is in agreement with our previous data showing chloride secretion induced by basolateral addition of Tat [16].

### Effects by gp120 on oxidative stress and apoptosis in Caco-2 cell monolayers

**Figure 8:**
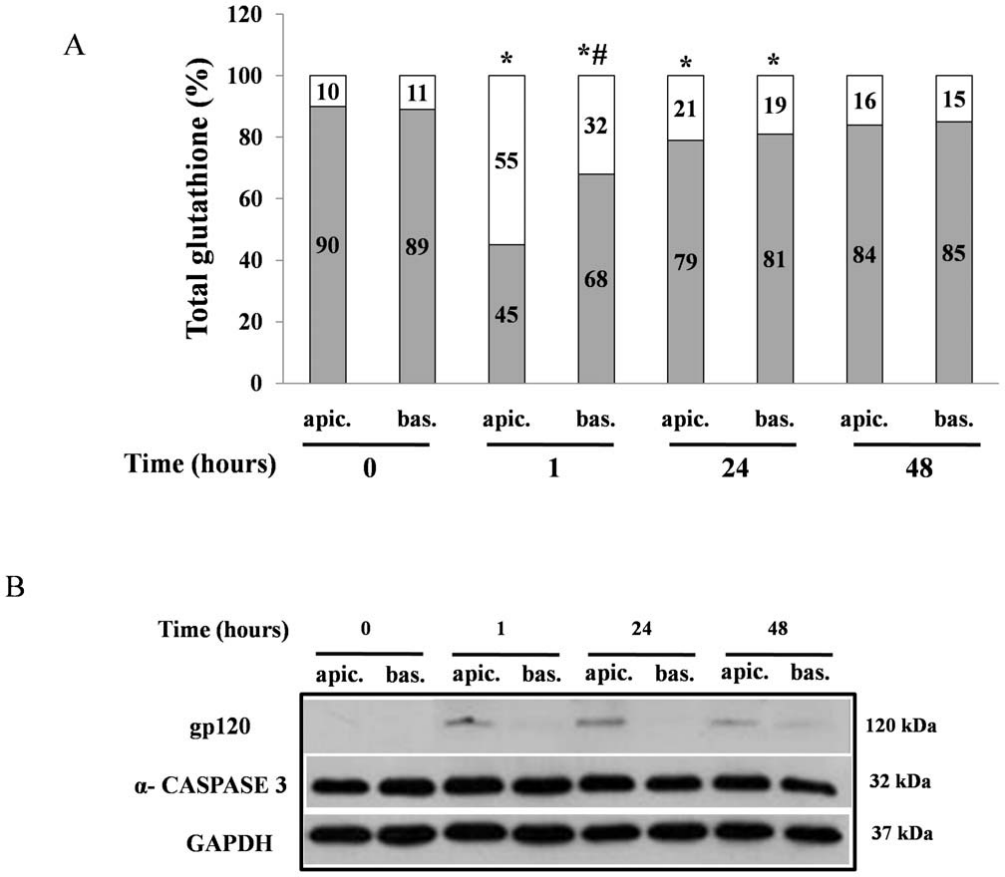
Effects by gp120 on oxidative stress and apoptosis in Caco-2 cell monolayers. gp120 (0.8 nM) was added to apical or basolateral side of Caco-2 cell monolayers and GSH/GSSG ratio (A) and caspase-3 activation (B) were evaluated after 1, 24 and 48 hours. *p<0,05 vs control; #p<0,05 vs apical gp120 stimulation at the same time. After gp120 stimulation cells were collected and western blots were performed with anti-gp120 polyclonal antibody (upper panel) and anti-caspase-3 monoclonal antibody (middle panel). Normalization of western blot was performed with GAPDH in all experiments (lower panel).

Previous studies investigating the effects of HIV-1 on epithelial barrier function, demonstrate that both Tat and gp120 (the latter being a surface envelope glycoprotein) directly increase permeability of brain endothelial cells through a redox-dependent mechanism [23], [30]. In order to investigate the similarities and differences between the effects induced by gp120 and by Tat, we evaluated the effects induced by gp120 on oxidative stress and apoptosis in our experimental system. An imbalance in GSH/GSSG ratio was observed in response to gp120 with a more potent effect at apical than basolateral side (Fig. 8A). However, gp120 did not induce caspase-3 activation (Fig. 8B).

### Tat-induced oxidative stress and apoptosis are strongly inhibited by pretreatment with antioxidants

**Figure 9:**
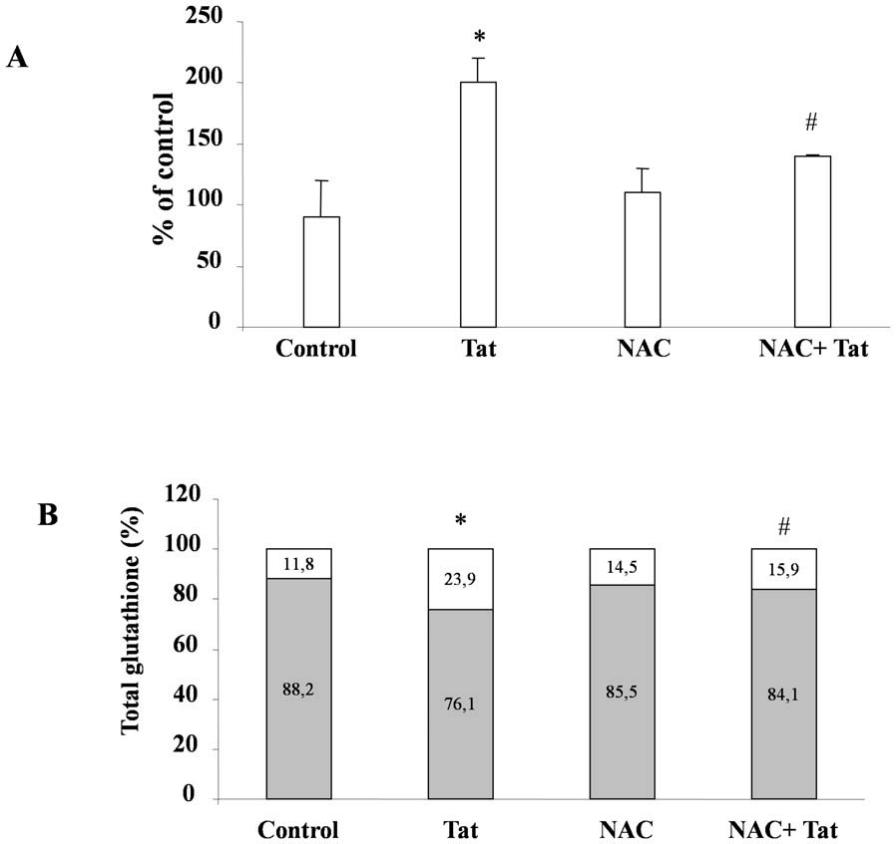
Effect of NAC on the Tat-induced oxidative stress in Caco-2 cells. Intracellular ROS levels, determined by fluorometric method, after exposure of Tat with or without pretreatment with NAC (A). Data were represented as percent of controls. Effect of NAC on Tat-induced GSH/GSSG imbalance (B). Data are represented as percent of GSH (grey) and GSSG (white) vs total glutathione. *p<0.05 vs control; #p<0.05 vs Tat. Data are representative of 3 separate experiments.

Our findings demonstrate that Tat induces oxidative stress and apoptosis in human enterocytes. These two events may be causally related in several pathogenic conditions [36]. We therefore used the antioxidant NAC to determine the relationship between oxidative stress and apoptosis induced by Tat at intestinal level. Our results demonstrate that oxidative stress is completely prevented by pretreatment with NAC. In fact, NAC prevented the Tat-induced ROS increase (Fig. 9A) and preserved the GSH/GSSG ratio (Fig. 9B).

**Figure 10:**
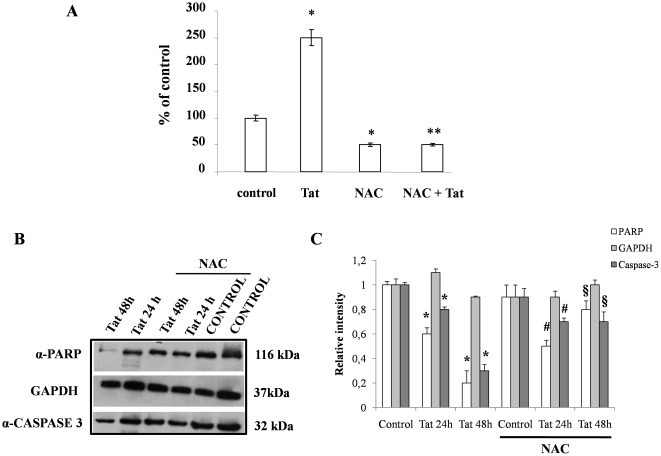
Effect of NAC on the Tat-induced apoptosis in Caco-2 cells. Caspase-3 activity (A) and full-length protein (B lower panel) were evaluated after exposure of Tat with or without pretreatment with NAC. Data were represented as percent of controls. *p<0,05 vs control; **p<0,05 vs Tat. Cleaved PARP was evaluated in the same western blot (B upper panel). Normalization of western blot was performed with GAPDH in all experiments (B middle panel). Data are representative of 3 separate experiments. (C) Densitometric acquisitions are shown from three separate experiments. *p<0,05 vs control; #p<0,05 vs control with NAC; §p<0,05 vs Tat at the same time without NAC.

To verify that the redox imbalance induced by Tat was the major cause of cell apoptosis, we performed experiments under a condition of oxidative stress prevention. Pretreatment for 24 hours with NAC completely prevented caspase-3 activity induced by Tat (Fig. 10A), suggesting that the redox imbalance caused apoptosis in our experimental model. We also evaluated caspase-3 and PARP cleavage by western blot and found that NAC prevented activation of caspase-3 signaling (Fig. 10B). Similar results were found in HT-29 cell line (Fig. S4). These results support the hypothesis that oxidative stress induced by Tat induces programmed cell death.

### Intestinal oxidative stress and apoptosis are related and prevented by the antioxidant NAC in human intestinal specimens

**Figure 11:**
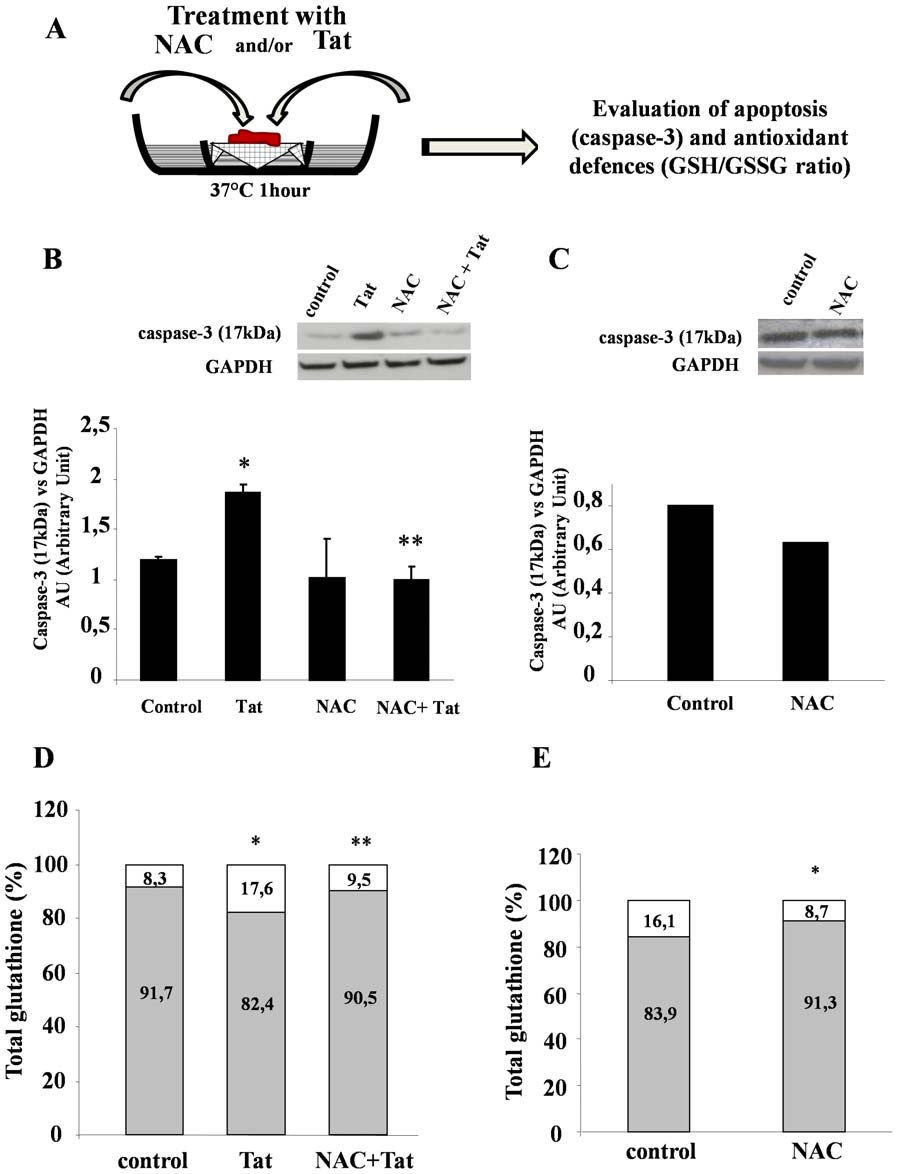
NAC prevented apoptosis and oxidative stress induced by Tat in human intestinal mucosa. Duodenal mucosal specimens were exposed to Tat alone or combined with NAC in an *ex-vivo* organ culture model (A). Panels B and C, Western blot on whole cell lysate was performed using anti-caspase-3 mouse monoclonal antibody (upper panel) and anti-GAPDH mouse monoclonal antibody in 4 HIV-negative children (B) and in an HIV-positive child (C). Densitometric acquisition of caspase-3 protein was normalized for GAPDH expression. Panels D and E, The GSH (grey)/GSSG (white) ratio was evaluated as described under “Methods” in intestinal mucosa of 4 HIV-negative children (D) and in an HIV-positive child (E). *p<0.05 vs control; **p<0.05 vs Tat. Results are expressed as the means SEM.

To determine whether the findings observed in Caco-2 cells were reproduced in human intestine, we evaluated apoptosis and redox intracellular homeostasis in duodenal biopsies from children (Fig. 11A). Western blot analysis for caspase-3 was performed on human intestinal specimens obtained from 4 HIV-negative patients. Similar to the findings we obtained in *in-vitro* cell models, exposure of human duodenal mucosa to Tat resulted in caspase-3 activation, as shown by the increase of the proteolytic 17-kDa fragment from caspase-3. Similar to the results we obtained in Caco-2 cells, this process was prevented by NAC (Fig. 11B).

Duodenal biopsies were obtained also from a single HIV-positive child to evaluate apoptosis and redox mechanisms in an *ex-vivo* model. Caspase-3 activation was determined in basal conditions and in the presence of NAC. Caspase-3 activation in specimens from the child was prevented by NAC (Fig. 11C).

Finally, we measured the GSH/GSSG ratio in intestinal biopsies. The ratio was decreased upon Tat exposure and the imbalance was prevented by NAC pretreatment in intestinal mucosa of HIV-negative patients (Fig. 11D). Interestingly, the baseline GSH/GSSG ratio in the duodenal tissue of an HIV-positive child (Fig. 11E) was similar to that observed in HIV-negative mucosa treated with Tat (Fig. 11D). However, it decreased after treatment with NAC and became similar to HIV-negative controls. These data strongly suggest that intestinal glutathione modifications are directly related to apoptosis in HIV-patients and that Tat, which was prevented by antioxidant treatment in human intestine, plays a key role in this condition.

## Discussion

Here we demonstrate that HIV-Tat protein directly induces enterocyte apoptosis by a redox-dependent mechanism. Our findings indicate that the increase in ROS intracellular level and impaired antioxidant defenses are associated with epithelial cell apoptosis and actin destruction. The effect was specific for Tat and the timing of the events suggested that Tat induced early oxidative stress followed by apoptosis. The oxidative stress was dose- and time-dependent reaching its peak at a concentration of 0.1 nM. This concentration is similar to that found in sera from HIV patients [37]. We also show that the antioxidant NAC is able to prevent intestinal oxidative stress by maintaining ROS and GSSG intracellular concentrations at a low level. In addition, NAC counteracted intestinal apoptosis, which supports the hypothesis that this event depends on a redox mechanism. Further support to this hypothesis comes from our evaluation of the redox balance and apoptosis in an *ex-vivo* experimental model. Caspase-3 cleaved protein was increased and the GSH/GSSG ratio imbalanced in intestinal biopsies exposed to Tat, which indicates that the apoptotic mechanism was triggered by the impairment of antioxidant defenses. Preincubation with NAC prevented these events with a pattern similar to that observed in cell lines. We had the opportunity to examine an intestinal specimen from an HIV-positive patient. Although we are aware that one HIV-infected patient is insufficient to make any conclusion, we found that apoptosis and the GSH/GSSG ratio in this sample were similar to those in HIV-negative biopsies exposed to Tat. Under these conditions, NAC decreased the levels of activated caspase-3 and re-established a GSH/GSSG ratio similar to those recorded in biopsies of HIV-negative patients. These data demonstrate that a redox-dependent mechanism is involved in the pathophysiology of HIV at intestinal level thereby implicating oxidative stress in HIV enteropathy. It would be interesting to detect Tat in the lamina propria and correlate the viral protein levels with those used in our experimental model. However, several authors detected HIV-1 RNA and viral antigens in epithelial intestinal cells in different stage of infection and in different type of cells [13], [14], [38]. Therefore it was suggested that the gut mucosa is a reservoir of HIV-1 infection and it is likely to think that viral proteins, including Tat, are present locally.

It has recently been demonstrated that the redox state of HIV-Tat affects its biological activity [39]. In particular, the Tat protein enters the cell and its reduced isoform reaches the cytoplasm and nucleus whereas its oxidized form tends to form multi-aggregates that are less toxic in infected cells. This suggests that the increase in intracellular ROS is not directly induced by Tat but is rather a strategy used by the infected cell to reduce the biological activity of Tat – a process that ultimately leads to programmed cell death.

In clinical studies, the redox balance was severely deranged in HIV-positive patients without highly active antiretroviral therapy (HAART) [40]. These studies showed that HIV-infected individuals are exposed to chronic exogenous oxidative stress that causes perturbations of the antioxidant defense system, including glutathione, thioredoxin, superoxide dismutase, ascorbic acid, glutathione peroxidase, tocopherol and selenium. In addition, hydroperoxidases and elevated levels of malondialdehyde were found in both pediatric and adult patients [41].

We first detected oxidative stress at intestinal level in HIV infection induced directly by Tat in cell models and in human intestinal epithelium. A marker of oxidative stress was also increased in rectal dialysis fluid, but not in serum and urine, of HIV-positive patients seen at our clinical center, which suggests that the stress event is localized at intestinal level. In addition, we found a significant correlation between viral load and concentration of 8-OHdG. We suggest that a specific clinical trial be conducted to evaluate intestinal damage and the therapeutic use of antioxidants.

We previously reported that Tat induces Ca^2+^-dependent chloride secretion in human intestinal cells [15]. Subsequently, we found that Tat inhibits glucose absorption in Caco-2 cells, and this effect was associated with SGLT-1 missorting [42]. Also α-tubulin staining was drastically decreased in Tat-exposed Caco-2 cells [42], which coincides with the altered actin structure we observed in Tat-treated Caco-2 and HT-29 cells. It was previously reported that Tat affects the microtubule cytoskeleton inducing apoptosis of T cells [43], [44]. Similar to what observed in T cells, Tat induces oxidative stress and destroys the cytoskeleton in intestinal cells and this is the result of the direct interaction with the enterocyte. Our findings show a profound disruption of the enterocyte cytoskeleton in HIV infection. Therefore, Tat induces a combined enterotoxic and a cytotoxic effect in human intestine.

The data reported herein were obtained in the same experimental model previously used to evaluate the enteropathogenic effects of Tat [16], [42] and show that the redox mechanism is implicated in the enteropathogenic effects of HIV. Tat-induced apoptosis in enterocytes is supported by our finding of the mitochondrial release of cytochrome c in the cytosol, the consequent caspase-3 activation and by increased PARP cleavage. All these effects are well in agreement with our previous result that Tat inhibits enterocyte cell proliferation [16], [42].

Attene-Ramos and colleagues demonstrated that the mucosal redox state is linked with intestinal proliferative activity [45]. Proliferating Caco-2 cells are vulnerable to oxidizing agents and a decrease in GSH levels induces transition to an apoptotic phenotype [46]. Moreover, an intracellular redox imbalance results in abnormal expression of genes involved in cell proliferation and growth, cell cycle progression, cytoskeleton structure and cell-to-cell adhesion [47], suggesting that redox alterations impair the enterocyte cycle. We found that Tat significantly induces oxidative stress and apoptosis in human intestinal epithelial cells with a specific sequence of events. Tat induced an early increase of ROS and an imbalance of the GSH/GSSG ratio that resulted in apoptosis. The two events are sequential and linked since an antioxidant prevented apoptotic cell damage.

Previously Nazli et al. demonstrated that gp120, but not Tat, affects intestinal epithelial integrity in a short time evaluation, with a major effect at apical side of intestinal epithelium [15]. Our results indicate that gp120 induces an imbalance in GSH/GSSG ratio with a more potent effect at apical side after 1 hour of stimulation but it does not induce significant caspase-3 activation suggesting that apoptosis is not induced by this viral protein. According to the different effects induced by Tat and gp120, we hypothesize that the two viral proteins act in an integrated mode with a different timing. gp120 is responsible to damage intestinal epithelium by luminal side in the early phase of infection allowing the virus to pass through the intestinal barrier. Subsequently Tat induces a sustained epithelial damage and oxidative stress which persists in the long term. These results raise the possibility of a novel therapeutic strategy for HIV patients, i.e., restoration of GSH levels. Antioxidants are considered therapeutic agents in oxidative stress-related diseases. Preliminary data indicate that NAC improves immunological functions and GSH levels in HIV patients [48], [49]. Our experimental results with NAC support this concept at intestinal level.

**Figure 12:**
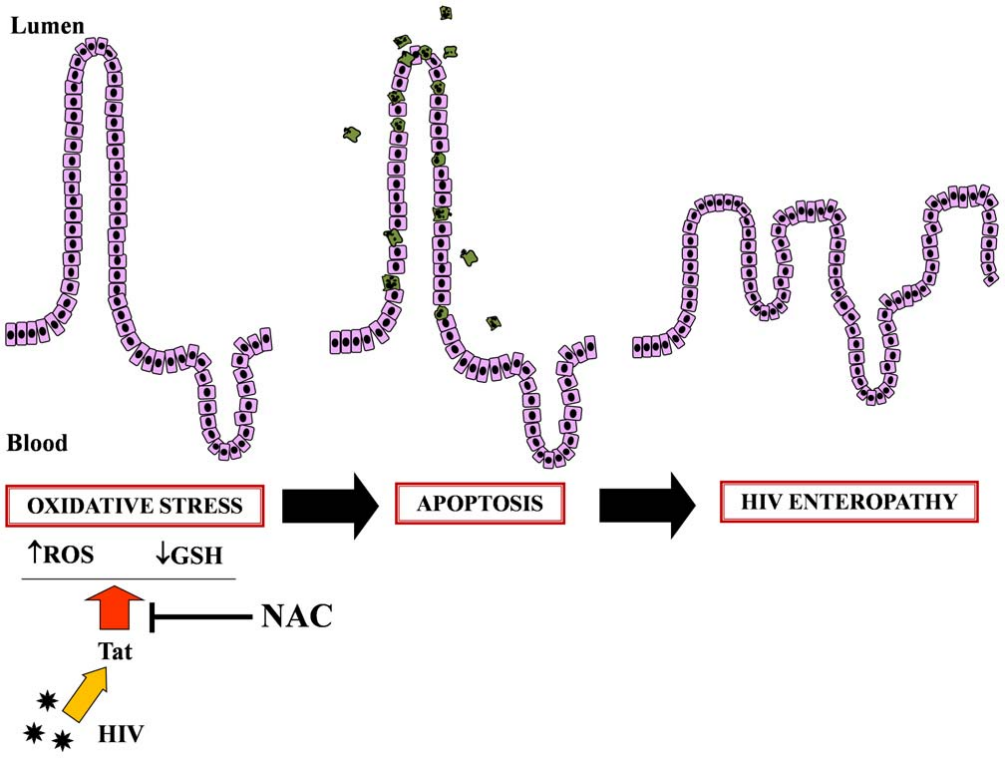
Schematic representation of the mechanism of HIV Tat viral protein-induced oxidative damage to the intestinal mucosa and the protective role of the antioxidant N-acetylcysteine (NAC). Tat induces oxidative stress by increasing the ROS intracellular level and deranging the GSH/GSSG ratio. This leads to programmed cell death (apoptosis) and an increase in epithelial damage. Together with ion secretion [15] and altered glucose transport [39], these steps could represent key mechanisms in HIV enteropathy. Pretreatment with the antioxidant NAC restores the oxidative stress and cell apoptosis, thus protecting intestinal mucosa from gut dysfunction.

In summary, the results of this study indicate that Tat induces oxidative stress by increasing ROS levels and impairing the GSH/GSSG balance in human intestinal epithelial cells suggesting a possible role in the HIV-enteropathy (Fig. 12). Tat also increases apoptosis. There is evidence that the intestinal tract, being a major lymphoid organ, is a source of virus replication [50]. Chun et al. have recently shown, in ileum biopsies from 8 patients, that gut-associated lymphoid tissue remains a major reservoir of HIV with potential residual cryptic replication despite long-term HAART [50]. In addition, HIV detection in gut mucosa is not influenced by levels of plasma viral load or antiretroviral therapy [13]. In this context there is a significant association between CD8+ T-cell response in rectal mucosa and plasma viral load and blood CD4 count in HIV-infected patients [51], [52]. The afore-mentioned results are fully in keeping with the concept that the intestine plays a major role in the pathogenesis of HIV infection, even in patients on successful HAART, and that Tat protein could be a major mediator of viral effects. Here we show that HIV-Tat protein plays a key role in inducing intestinal functional alterations including transepithelial secretion, glucose malabsorption and finally an oxidative stress condition with apoptosis leading to a compromised epithelial architecture and pretreatment with the antioxidant NAC prevented redox imbalance and apoptosis.

## Methods

### Intestinal cell line cultures

Caco-2 cells were used as small intestinal cell model [53]. At 15 days post-confluence, cells exhibit a well-differentiated brush border on the apical surface and tight junctions with typical small-intestinal microvillus hydrolases and nutrient transporters. Caco-2 cells were grown in Dulbecco's modified Eagle minimum essential medium (DMEM; Gibco, USA) with a high glucose concentration (4.5 g/L) at 37°C in a 5% CO_2_ atmosphere. The medium was supplemented with 10% fetal bovine serum (FBS, Gibco, USA), 1% non-essential amino acids, penicillin (50 mU/mL) and streptomycin (50 µg/mL), and changed daily. To verify some data obtained in Caco-2 cells, we used HT-29 as a supplementary intestinal cell model. HT-29 were grown in RPMI 1640 cell culture medium supplemented with 10% fetal bovine serum (FBS, Gibco, USA), 100 mU/ml penicillin and 100 µg/ml streptomycin.

To evaluate the polar nature of the effects induced by Tat on Caco-2 cells, monolayers were grown on polycarbonate filter (Corning Incorporated, NY USA) for 15 days post-confluence and then exposed to Tat 0,5 nM for 1, 24 and 48 hours at apical or basolateral side and then GSH/GSSG ratio and caspase-3 activation were evaluated as previously described. Controls were stimulated with the same volume of medium without Tat.

### Fluorescence microscopy

Cells were grown on the chambered cover glass for 3 days. Cells were exposed to 20 µM 7′-dichlorofluorescein diacetate (DCFH-DA, D6665; Sigma-Aldrich, St. Louis, MO) for 1 hour at 37°C in the dark. Then, they were treated with Tat (0.5 nM), adding the protein directly to the cell culture, and incubated at 37°C for 1 hour to detect the ROS. Cells were washed in PBS and after mounting, the fluorescence images from multiple fields of view were obtained using a Nikon Eclipse 80i microscopy. The images were analyzed using NIS Elements D imaging software. A positive control was obtained by incubating cells with H_2_O_2_ 10 mM for 5 min.

### Reactive oxygen species production

The production of ROS was measured using the DCFH-DA fluorometric method, which is based on the ROS-dependent oxidation of DCFH-DA to DCF. Caco-2 cells were grown in 6-well plates for 15 days post-confluence. Cell monolayers were treated with Tat 0.05, 0.1 and 1 nM for 15 min or with Tat 0.5 n M for 15, 30, 60 and 120 min at 37°C. Medium was removed and cells were washed by PBS. Then, cells were treated with DCFH-DA (20 µM) for 30 min at 37°C in the dark. Intracellular ROS production was measured with a spetrofluorometer (SFM 25; Kontron Instruments, Japan). A positive control was obtained by incubating cells with H_2_O_2_ 10 mM for 5 min. Neutralization experiments were performed incubating Tat 0.5 nM with 30 ng/ml of anti-Tat polyclonal antibodies (Tecnogen, Piana di Monte Verna, Italy) at +4°C overnight with gentle shaking. The preparations were then used to stimulate cells.

### Antioxidant defenses evaluation

GSH and GSSG intracellular levels were measured by method described by Allen et al. with few modifications [54]. Briefly, cells were exposed to Tat alone or in combination with NAC and then lysed with Triton X-100. Protein was precipitated with 1% sulfosalicylic acid and supernatants used to measure in parallel total and reduced glutathione. Oxidated glutathione (GSSG) was determined by subtracting the reduced form from total glutathione. GSH and GSSG values were normalized for protein content. All assays were performed three times. Catalase activity, a well-established indicator of oxidative stress, was measured with an assay kit from Calbiochem (Gibbstown, NJ) and normalized for protein content. Neutralization experiments were performed incubating Tat 0.5 nM with 30 ng/ml of anti-Tat polyclonal antibodies (Tecnogen, Piana di Monte Verna, Italy) at +4°C overnight with gentle shaking. The preparations were then used to stimulate cells. gp120 (ab69717) and goat polyclonal anti-gp120 antibody (ab21179) were from Abcam plc (Cambridge, UK).

### Oxidative stress evaluation in HIV-positive patients

An aliquot of rectal dyalisis, urine and serum samples from 20 patients infected with HIV and 6 control subjects aged 26–228 months were obtained during a routine check-up. All HIV patients had been on HAART for more than 3 years. Immediately after collection, samples were separated into 2 mL aliquots and stored at −80°C until analysis. We used levels of 8-hydroxy-2′-deoxyguanosine (8-OHdG), as biomarker of DNA oxidative stress. 8-OHdG was measured in duplicate using a highly sensitive ELISA kit (Li StarFish Srl, Milan, Italy). The length of dialysis bag is 3 cm and the concentration of oxidative marker was normalized for the exact volume inside the bag. Differences were evaluated by the Mann-Whitney test. The Pearson's correlation coefficient was applied to investigate the correlation between 8-OHdG and viral load.

### Actin staining

One step of fixation and permeabilization was performed with 4% paraformaldehyde and 0.2% Triton X-100 for 30 min at +4°C. After three washes in PBS, the cells were treated with a 50 µg/ml solution of fluorescein isothiocyanate-phalloidin (Sigma-Aldrich, Milan, Italy) in PBS for 40 min. Nuclei were stained with Hoechst 5 µg/ml (Sigma-Aldrich, Milan, Italy) for 5 min at +4°C. The cells were washed three times with PBS and were mounted with Mowiol (Invitrogen S.R.L, San Giuliano Milanese, Italy). The monolayers were examined using a Nikon Eclipse 80i epifluorescent microscope (FITC filter). The images were analyzed using NIS Elements D imaging software.

### Caspase-3 activity assay

We used caspase-3 as a marker of apoptosis [55]. An apoptosis assay kit was used to determine caspase-3 activity, according to the manufacturer's instructions (Biovision, Mountain View, CA). Caspase-3 activity was investigated in Caco-2 cells by the release of the chromophore pNA after substrate cleavage. Modifications of caspase-3 activity were determined by comparing the sample optical density (OD) with the control.

### Immunoblotting

Total cells lysates were obtained by homogenization of cell pellets in cold lysis buffer (20 mM Tris, pH 7.5 containing 300 mM sucrose, 60 mM KC1, 15 mM NaC1, 5% (v/v) glycerol, 2 mM EDTA, 1% (v/v) Triton X-100, 1 mM PMSF, 2 mg/ml aprotinin, 2 mg/ml leupeptin and 0.2% (w/v) deoxycholate) for 1 min at 4°C and further sonication for additional 30 sec at 4°C. Cytosolic, microsomal and mitochondrial fractions were prepared with the Qproteome Mitochondria Isolation Kit (Qiagen). Equal amounts of protein were subjected to 10% (v/v) SDS-PAGE and transferred to a PVDF membrane (Millipore). The membrane was blocked with 5% (w/v) skim milk and incubated with primary antibody, followed by incubation with an HRP-conjugated secondary antibody. Proteins were visualized with an ECL detection system (GE-Healthcare). The following antibodies were used for Western blot analysis: rabbit polyclonal anti-Tat antibody (Tecnogen, Piana di Monte Verna, Italy), mouse monoclonal anti-caspase3 antibody (full length protein), mouse monoclonal anti-PARP antibody, goat polyclonal cytochrome c antibody, mouse monoclonal anti-COX IV antibody, mouse monoclonal anti-tubulin antibody, mouse monoclonal anti-GAPDH antibody (Santa Cruz Biotechnology). gp120 (ab69717) and goat polyclonal anti-gp120 antibody (ab21179) were from Abcam plc (Cambridge, UK).

### Co-immunoprecipitation

A total of 800 µg of protein lysate from the Caco-2 cells exposed to Tat 0,5 nM 24 hrs were precipitated with 1 mg of tubulin antibody. Protein A/G agarose beads (Santa Cruz) were used to collect the immunoprecipitated complexes and the beads were washed with PBST before SDS-PAGE and Western blot analysis with anti-Tat antibody.

### Experiments in human small intestinal specimens

Biopsies from the distal part of the duodenum were obtained from 5 children seen at the Department of Pediatrics and undergoing endoscopy (84–192 months of the age) for intestinal disorders. All biopsies were from macroscopically normal areas, and intestinal histology was subsequently reported to be normal. Tissue samples were transported to the laboratory in culture medium and processed within one hour. Duodenal mucosa specimens were obtained from 4 HIV-negative children and from one HIV-positive child. Specimens were washed and observed by stereomicroscope to exclude tissue necrosis. Organ culture was performed in DMEM with a high glucose concentration (4.5 g/L) supplemented with 0.5% FCS, 1% non-essential amino acids, 2% penicillin (50 mU/mL) and streptomycin (50 mg/mL) and incubated in 5% CO_2_/95% air for one hour before treatment. Short-term experiments were run using high Tat concentrations to maximize the cytotoxic effect before spontaneous tissue disruption. Specimens were exposed to Tat alone (0.1 µM) or preincubated with NAC (10 mM for 4 h). Short-term experiments with an higher Tat concentration were performed to maximize the effect before spontaneous tissue disruption. After stimulation, samples were homogenized and lysed in RIPA buffer: 100 mM Tris-HCL pH 7.5, 300 mM NaCl, 2% NP40, 1% Na deoxycholic acid, 0.2% SDS, 100 µg/ml PMSF, 5 µg/mL aprotinin, 1 µg/mL leupeptin, 0.7 µg/ml pepstatin. Whole extracts were centrifuged and protein content was determined by the Bradford assay. GSH/GSSG intracellular levels and caspase-3-cleaved protein were evaluated as described above. For western blot assay we used the mouse monoclonal anti-caspase-3 cleaved protein (Cell Signaling Inc., Danvers, MA). The experiments were undertaken with the understanding and written consent of each child's parents and the study methodologies conformed to the standards set by the Declaration of Helsinki. The study protocol was approved by the Ethics Committee of the School of Medicine, University of Naples Federico II, Italy.

### Statistical analysis

We used GraphPad Prism Software (San Diego, CA) to evaluate the two-tailed unpaired Student *t* test and a 2-tailed paired Student *t* test to evaluate statistical significance. An alpha value of 0.05 was set for statistical significance. p-Values for each analysis are indicated in figure legends.

## Supporting Information

Figure S1 **Fluorescence staining of ROS.** ROS intracellular levels were evaluated in Caco-2 cells by DCF fluorimetric method (A) and at fluorescence microscope (B). In parallel, cells from the same culture and in the same conditions were exposed to Tat and a western blot was performed with anti-Tat polyclonal antibody (C). *p<0,05 vs control.
(TIF)

Figure S2 **The anti-Tat polyclonal antibody blocks the Tat-induced imbalance of the GSH/GSSG ratio in HT-29 cells.** HT-29 cells were incubated with Tat in the presence and absence of the anti-Tat polyclonal antibody. Data are represented as percent of GSH (grey) and GSSG (white) vs total glutathione. Data are representative of 3 separate experiments.*p<0,05 vs control; **p<0,05 vs Tat.
(TIF)

Figure S3 **8-OHdG levels in urine and serum in HIV-negative and -positive children.** 8-OHdG was used as oxidative stress marker evaluated in urine (A) and serum (B) in serum-negative (SN) and positive (HIV+) children. There were no significant differences between the two groups.
(TIF)

Figure S4 **Influence of HIV-Tat protein on apoptosis in HT-29 cells.** Caspase-3 activity (A) and full-length protein (B lower panel) were evaluated in Tat-treated HT-29 cells. To verify that Tat induced apoptosis, cleaved PARP was evaluated in the same western blot used to evaluate the activation of caspase-3 (B upper panel). Normalization of western blot was performed with GAPDH in all experiments (B middle panel). Data are representative of 3 separate experiments.
(TIF)
