# Supplementary figures and images for: The HIV-1 Transactivator Factor (Tat) Induces Enterocyte Apoptosis through a Redox-Mediated Mechanism
Source: PLoS One. 2011 Dec 27;6(12):e29436. doi: 10.1371/journal.pone.0029436 (PMC3246489; doi:10.1371/journal.pone.0029436)

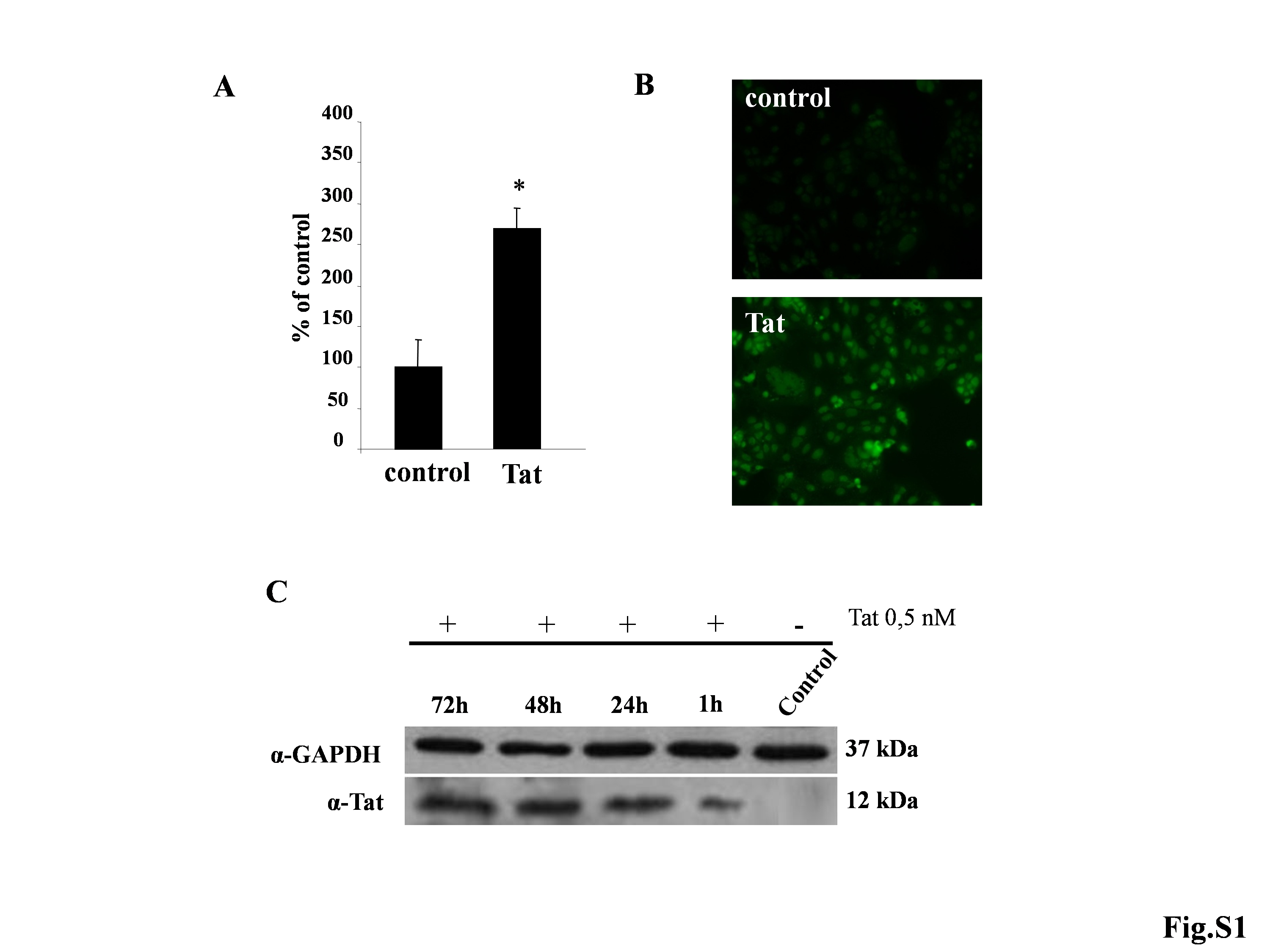

Supplement: Figure S1 — Fluorescence staining of ROS. ROS intracellular levels were evaluated in Caco-2 cells by DCF fluorimetric method (A) and at fluorescence microscope (B). In parallel, cells from the same culture and in the same conditions were exposed to Tat and a western blot was performed with anti-Tat polyclonal antibody (C). *p<0,05 vs control. (TIF) [file pone.0029436.s001.tif]

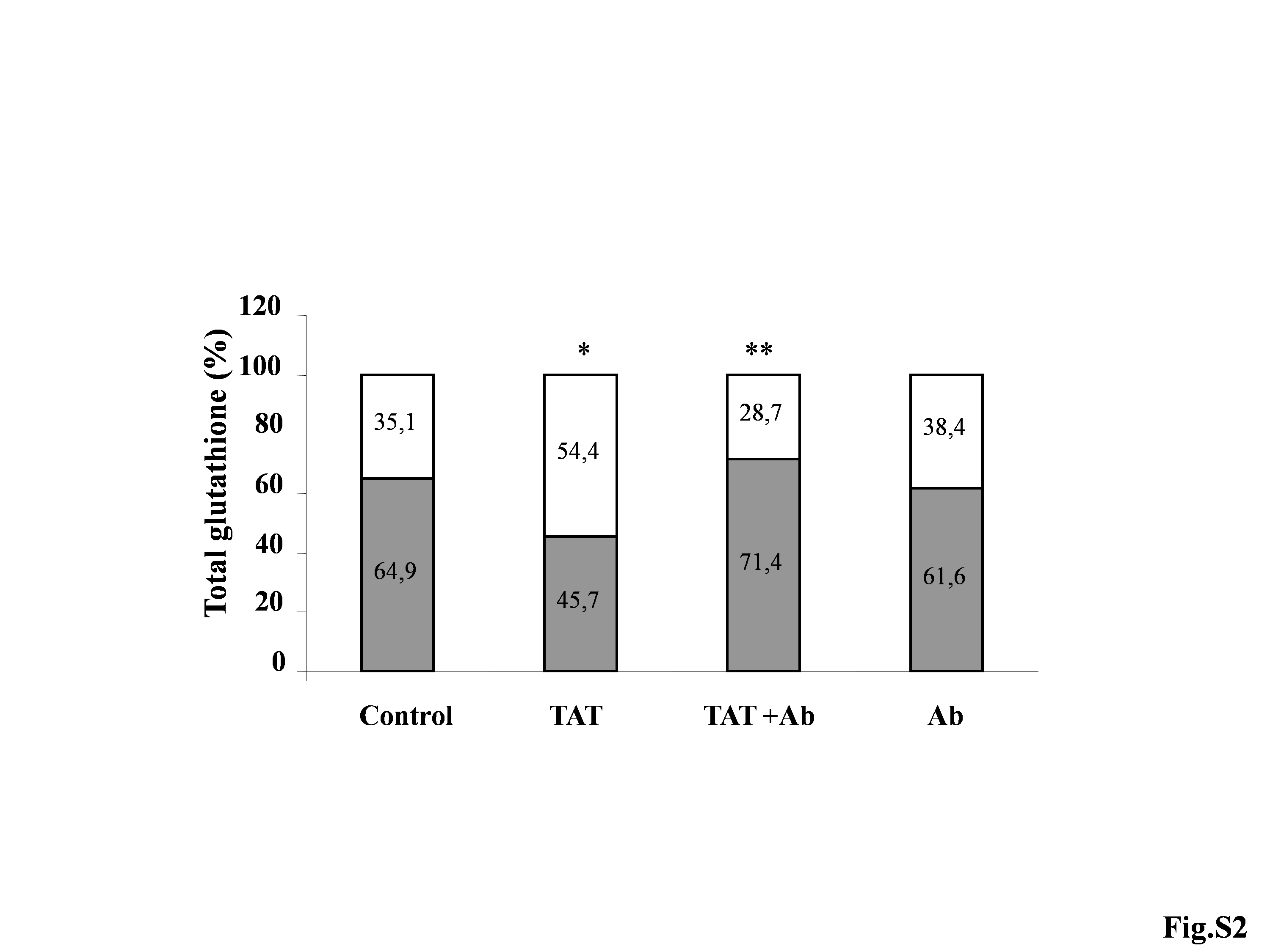

Supplement: Figure S2 — The anti-Tat polyclonal antibody blocks the Tat-induced imbalance of the GSH/GSSG ratio in HT-29 cells. HT-29 cells were incubated with Tat in the presence and absence of the anti-Tat polyclonal antibody. Data are represented as percent of GSH (grey) and GSSG (white) vs total glutathione. Data are representative of 3 separate experiments.*p<0,05 vs control; **p<0,05 vs Tat. (TIF) [file pone.0029436.s002.tif]

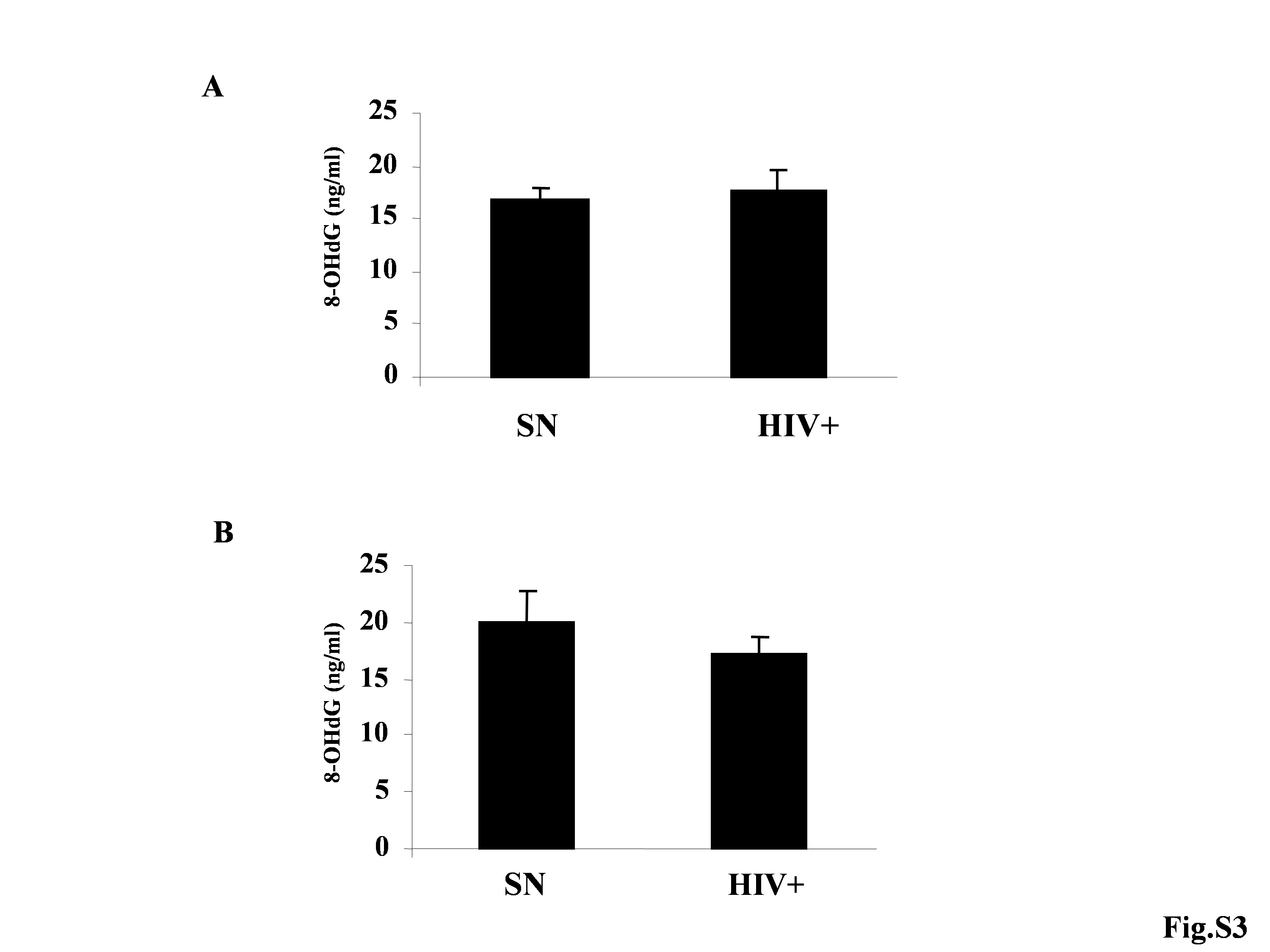

Supplement: Figure S3 — 8-OHdG levels in urine and serum in HIV-negative and -positive children. 8-OHdG was used as oxidative stress marker evaluated in urine (A) and serum (B) in serum-negative (SN) and positive (HIV+) children. There were no significant differences between the two groups. (TIF) [file pone.0029436.s003.tif]

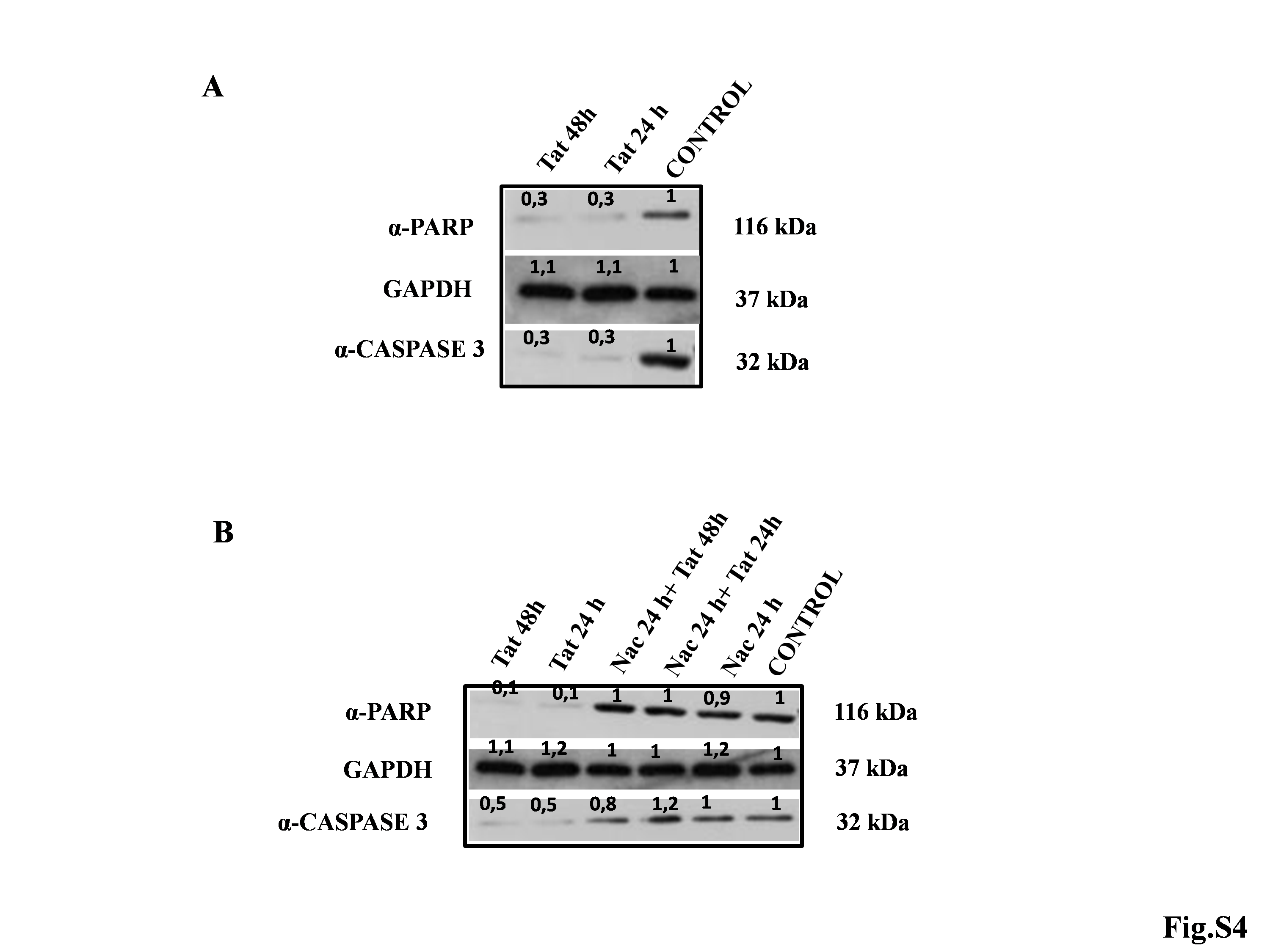

Supplement: Figure S4 — Influence of HIV-Tat protein on apoptosis in HT-29 cells. Caspase-3 activity (A) and full-length protein (B lower panel) were evaluated in Tat-treated HT-29 cells. To verify that Tat induced apoptosis, cleaved PARP was evaluated in the same western blot used to evaluate the activation of caspase-3 (B upper panel). Normalization of western blot was performed with GAPDH in all experiments (B middle panel). Data are representative of 3 separate experiments. (TIF) [file pone.0029436.s004.tif]
